# A scientometric analysis and visualization of kinesiophobia research from 2002 to 2022: A review

**DOI:** 10.1097/MD.0000000000035872

**Published:** 2023-11-03

**Authors:** Linzhang Li, Yan Sun, Hua Qin, Jun Zhou, Xiaojuan Yang, Aiying Li, Jin Zhang, Yan Zhang

**Affiliations:** a Wenjiang People’s Hospital of Chengdu, Chengdu, China; b School of Medicine, University of Electronic Science and Technology of China, Chengdu, China; c Sichuan Academy of Medical Sciences & Sichuan Provincial People’s Hospital, Chengdu, China; d The Philippines Women’s University, Manila, Metro Manila, Philippines; e School of Nursing, Chengdu University of Traditional Chinese Medicine, Chengdu, China.

**Keywords:** CiteSpace, kinesiophobia, scientometric analysis, visualization, VOSviewer

## Abstract

Kinesiophobia is an excessive, irrational, debilitating fear of physical movement and activity caused by a sense of vulnerability to pain or re-injury, which can have a direct impact on physical functioning and mental well-being of patients. This paper aims to provide reliable support for future in-depth research on kinesiophobia through scientometrics and historical review. Studies on kinesiophobia published from 2002 to 2022 were retrieved from the Web of Science Core Collection. CiteSpace and VOSviewer were used to conduct bibliometric analysis of the included studies and map knowledge domains. Keywords were manually clustered, and the results were analyzed and summarized in combination with a literature review. A total of 4157 original research articles and reviews were included. Research on kinesiophobia is developing steadily and has received more attention from scholars in recent years. There are regional differences in the distribution of research. Chronic pain is the focus of research in this field. A multidisciplinary model of pain neuroscience education combined with physical therapy based on cognitive–behavioral therapy and the introduction and development of virtual reality may be the frontier of research. There is a large space for the study of kinesiophobia. In the future, to improve regional academic exchanges and cooperation, more attention should be given to the clinical applicability and translation of scientific work, which will be conducive to improving the quality of life and physical and mental health outcomes of kinesiophobia patients.

## 1. Introduction

In 1983, Lethem et al^[1]^ proposed the “fear-avoidance” model, in which the central concept is the patient fear of pain. “Confrontation” and “avoidance” are thought to be the 2 extreme responses to this fear, with the former causing the patient to become progressively physically and socially active after the organic basis of pain has been eliminated, and fear is reduced over time. The latter can have a range of physiological and psychological consequences that promote the development of ineffective states and exaggerated pain perceptions, allowing fear to be maintained or exacerbated. In 1990, Kori et al^[2]^ introduced the term “kinesiophobia,” which refers to an excessive, irrational and debilitating fear of physical movement and activity caused by patients who feel vulnerable to painful injury or (re)injury. Cognitive-behavioral model of fear of move/(re)injury argues^[3]^ that when a painful experience is interpreted as a threat, it leads to the belief that exercise causes pain and risk of reinjury, leading to avoidance behavior and withdrawal from activities that may cause painful and uncomfortable situations. Knapik et al^[4]^ developed a new perspective by defining kinesiophobia as the fear of experiencing physical or psychological discomfort. The negative effects of kinesiophobia on patients are both physical and psychological. Over time, long-term avoidance or avoidance behaviors may lead to adverse outcomes such as disability, disuse and psychological stress^[5,6]^ and may also affect rehabilitation outcomes by weakening rehabilitation motivation and exercise compliance.^[7]^ Review of the literature revealed that in addition to patients with chronic back pain, who commonly experience kinesiophobia, other diseases accompanied by pain, such as Parkinson disease, fatigue, heart disease, temporomandibular joint disease, scoliosis, and even asthma and stroke may also lead to kinesiophobia.^[8–12]^ Due to the diversity and complexity of the risk factors and neurophysiological mechanisms of kinesiophobia,^[13,14]^ which have a profound impact on the physical and mental health and quality of life of patients,^[5,8]^ in-depth understanding and analysis of kinesiophobia remains a top priority in future work to develop more targeted and effective screening, prevention and treatment strategies. Ultimately, the health outcomes and quality of life of kinesiophobia patients may be improved, and the burden on family and society may be reduced.

To understand the knowledge base of kinesiophobia research in the past 20 years, identify research hot spots and the evolution of this field, explore potential trends of future research, and provide references and support for future research on kinesiophobia, we conducted a scientometric analysis of the field of kinesiophobia research. Maps of knowledge domains show the relationship between the development and the structure of scientific knowledge as the object of knowledge domains. These maps are not only a visualization but also a serialized knowledge pedigree, showing a large number of implied complex relations such as networks, structure, crossovers, evolution or derivation among knowledge units or knowledge groups, and these complex relations can give rise to new knowledge.^[15]^ CiteSpace and VOSviewer are popular software for mapping knowledge domains and have been widely used in scientometrics research.^[16,17]^ We mapped the country/region co-occurrence network and institution co-occurrence network with CiteSpace, conducted co-citation analysis and keyword burst analysis, and mapped the author co-occurrence network and keyword co-occurrence network with VOSviewer. The distribution, knowledge structure, research topics and trends of kinesiophobia research were revealed. Along with a historical review of artificial clustering results, we provide a comprehensive and objective analysis and summary of the topics discussed in this field in the current study.

## 2. Materials and methods

### 2.1. Ethic statement

Ethical approval is not required for this study because it is conducted based on secondary data.

### 2.2. Data source

**Figure 1.**
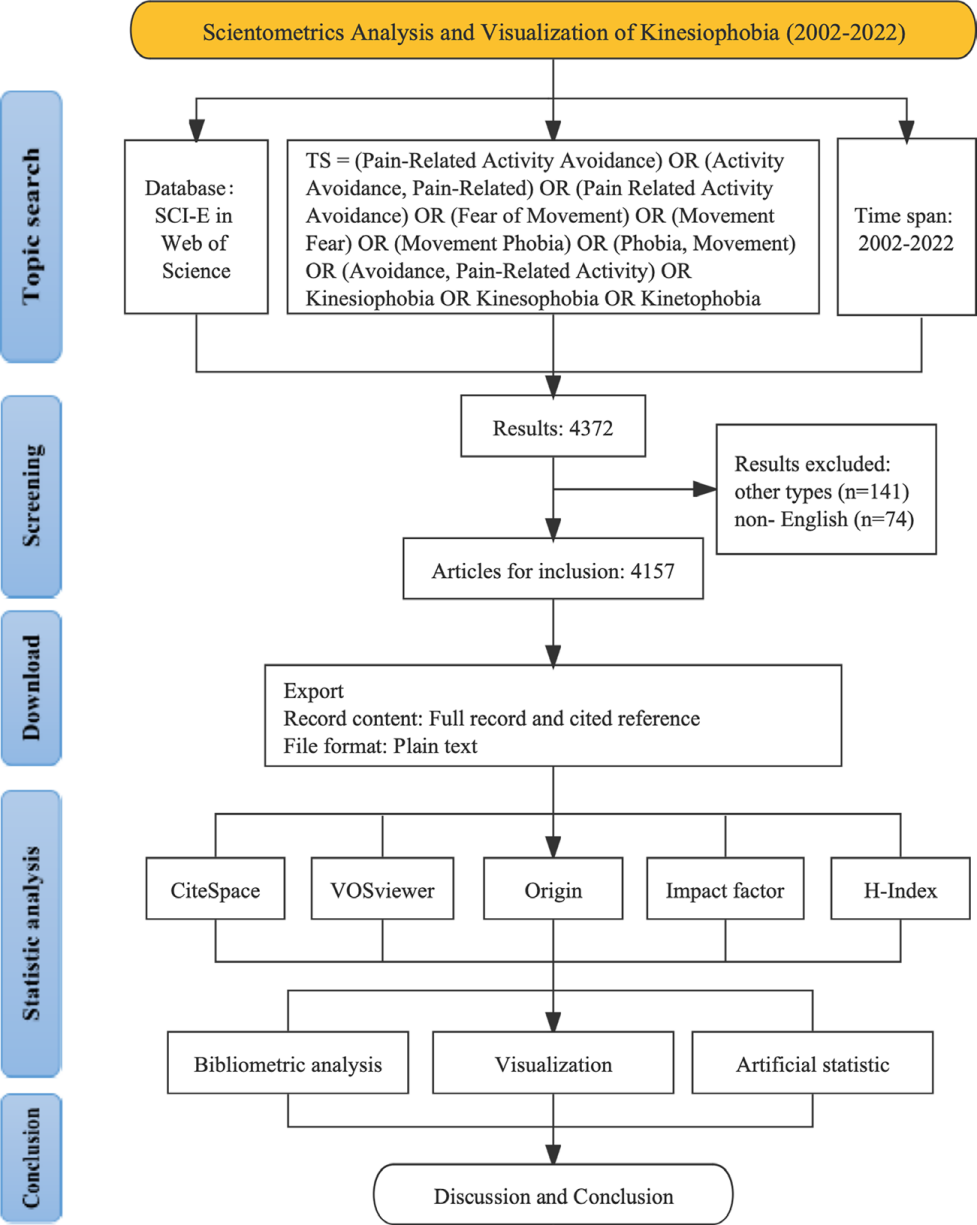
Flowchart of the scientometric analysis.

The citation data of this study were retrieved from the Web of Science Core Collection (WoSCC), Science Citation Index Expanded database (SCI-E). The topic search strategy was as follows: TS =((Pain-Related Activity Avoidance) OR (Activity Avoidance, Pain-Related) OR (Pain Related Activity Avoidance) OR (Fear of Movement) OR (Movement Fear) OR (Movement Phobia) OR (Phobia, Movement) OR (Avoidance, Pain-Related Activity) OR Kinesiophobia OR Kinesophobia OR Kinetophobia). The publication time range was as follows: 2002-01-01 to 2022-12-31. Other qualifications: Original article or reviews were included if published in English. Finally, a total of 4157 citation records were identified for inclusion. Files were downloaded in as plain text files, and the record content was “Full Record and Cited References.” In this way, the data were sorted for use with visual analysis software. The process of this study is shown in Figure 1.

### 2.3. Research methods

The bibliometric analysis was performed with the help of the visual analysis software CiteSpace 6.2.3 (64-bit) and VOSviewer 1.6.19 (0), both of which are applications written in Java.^[18,19]^ The items analyzed in this study include category, country/region distribution and institute distribution based on CiteSpace maps of knowledge domains, co-citations, keywords with citation bursts, and keyword co-occurrence based on VOSviewer, supplemented by artificial clustering and a historical review and discussion. Some parameters of CiteSpace were set as follows. The time slice was set at 2002 to 2022, and each year was considered 1 slice. The node type was selected from the items mentioned above. The selection criterion for each slice was as follows: Top N = 50 (i.e., select the top 50 levels of the most cited or cooccurring items from each slice). VOSviewer was run to read the downloaded bibliographic database file and create a map. “Author” and “author keywords” were selected in the cooccurrence analysis unit, and “full counting” was used as the counting method. In addition, the impact factor (IF) and H-index were introduced into the relevant analyses to ensure a comprehensive analysis of scientometric results.

## 3. Results

**Figure 2.**
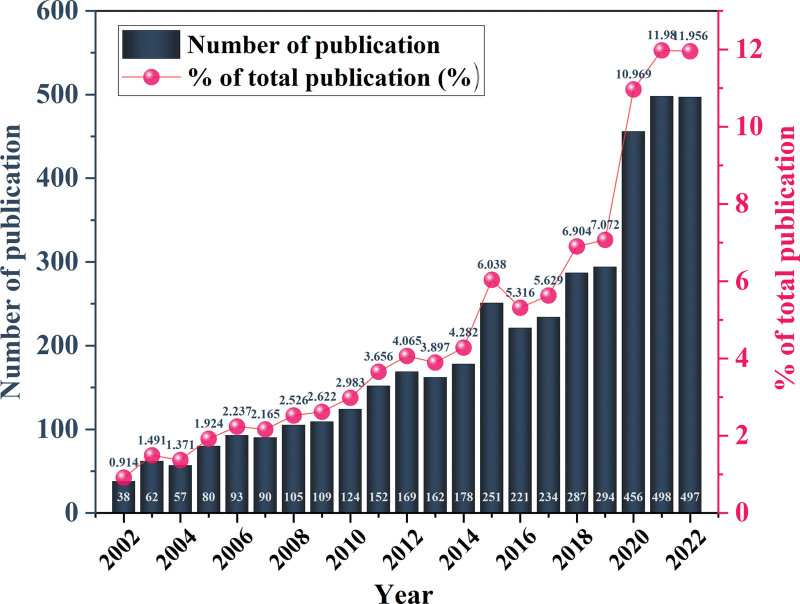
Annual publications (2002–2022).

Based on the citation data from the WOS database, the total number of publications on kinesiophobia has increased substantially in the past 20 years. Although there were slight fluctuations in growth, the overall trend is increasing (Fig. 2). The increases in 2015 and 2020 were particularly prominent, but the increase in 2015 was not sustained. After 2019, research on this topic achieved important breakthroughs, suggesting that kinesiophobia has received increasing attention from scholars in recent years. This may be because in contemporary society, people not only pay attention to physical health but have higher requirements for psychological and spiritual health. On the other hand, the fluctuation of the growth of this research area suggests that our research in this field may encounter certain difficulties, and more in-depth research is needed in the future to diagnose and understand kinesiophobia.

### 3.1. Country/region distribution

**Figure 3.**
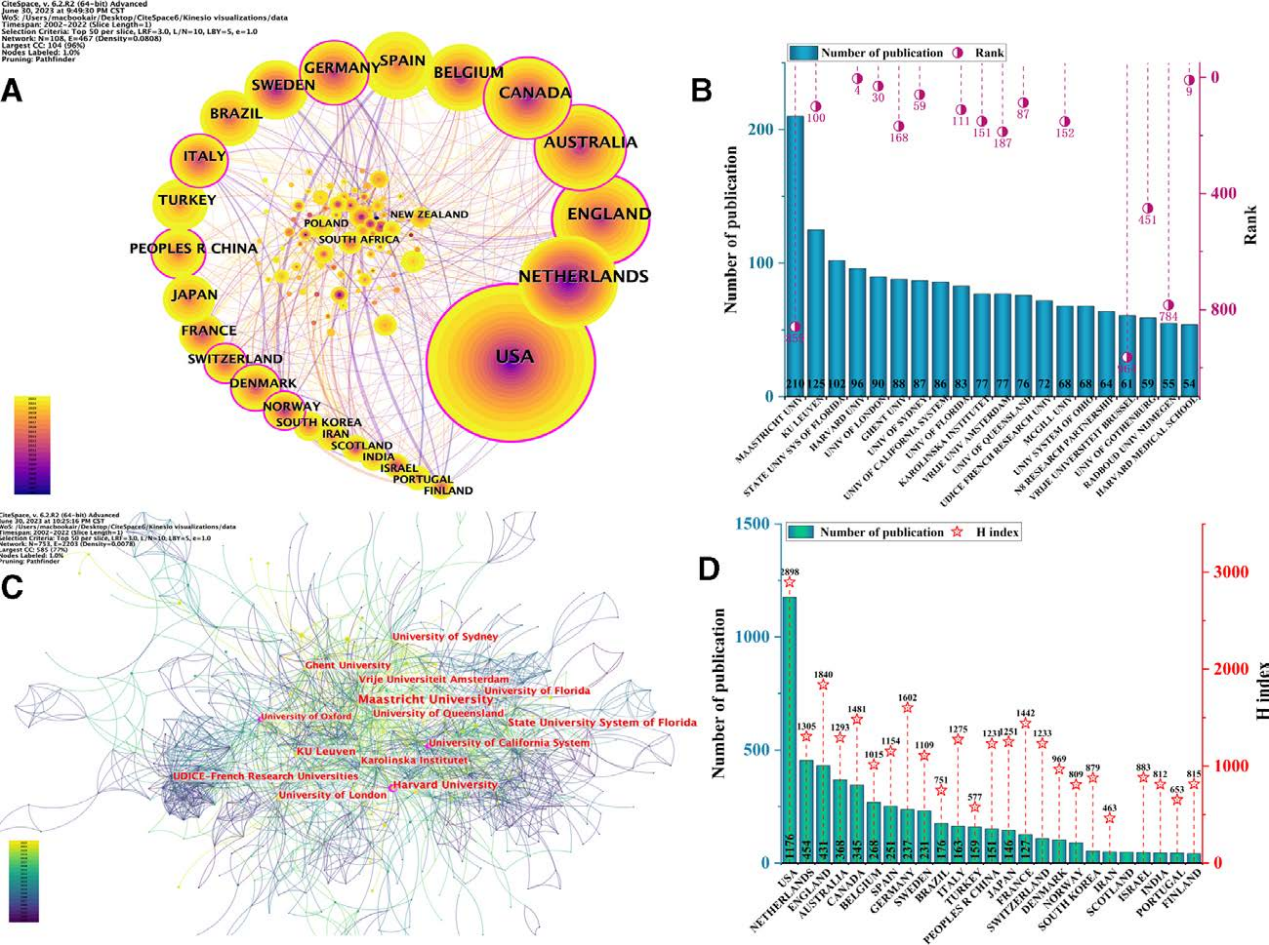
Cooperation of countries/regions and institutes. Mapping of country/region co-occurrence network. The size of node and line represent the number of publications from a country/region and the cooperation between countries/regions, respectively. The color of each layer of the node represents the corresponding year of publication, and the color and thickness of the lines indicate when the cooperation started and how close the cooperation is, respectively. (B) Number of publications and H-index for countries/regions. (C) Mapping of institute co-occurrence network. A node represents a institute, and the line represents the cooperative relationship in institutes. The color of the lines indicate when the cooperation started. The node whose outermost color is purple indicates a centrality >0.1. (D) Number of publication and global institutional rank.

We used CiteSpace to obtain the co-occurrence network of countries/regions, visualizing the cooperation relationships between countries/regions (Fig. 3A). Each node represents a country, the size of the node represents the number of publications of that country, and the connecting line between the nodes indicates cooperation between the 2 countries. The information in Figure 3A shows that research on this topic was concentrated in North America, Europe and Oceania (specifically, Australia). The United States ranked first in the number of publications (1176, 28.29% of the total), followed by the Netherlands, England, Australia, Canada, Belgium, Spain, Germany, Sweden, and other regions (Brazil, Japan, China, New Zealand, etc). The number of publications in the Netherlands was 454, accounting for 10.921% of the total; the number of publications in the England was 431, accounting for 10.392% of the total; the number of publications in China was 151, accounting for 3.631% of the total; and the number of publications in Japan was 146, accounting for 3.512% (Fig. 3B). The research contribution of the United States in this field was several times ahead of other countries and regions. Worldwide, the distribution of kinesiophobia studies among countries was unbalanced. The H-index can reflect the academic achievement and influence of a subject. The knowledge map of the cooperation network among countries/regions revealed relatively dense connections among nodes (density = 0.0808) and thus a certain degree of cooperation among countries/regions.

### 3.2. Institute distribution

The knowledge map of the institutional co-occurrence network (Fig. 3C) had 753 nodes, 2203 edges, and a density = 0.0078, indicating that numerous research institutions have conducted related research on kinesiophobia. Nodes with centrality >0.1 play a key role in these networks and are represented in Figure 3C by nodes in the purple outer layer. Specifically, the institutions with strong centrality in kinesiophobia research were Harvard University, the University of Oxford, and the University of California system. Moreover, among the top-ranked institutions in terms of total publications, there were world-famous institutions and highly influential university systems, such as Harvard University, the University of London, the University of Sydney, Harvard Medical School, and the State University System of Florida (Fig. 3D), all of which are organizations with top scientific research strength and level. Therefore, we can expect further important results in this field in the future. Additionally, Europe and the United States were in the leading position in this research field, followed by Australia. However, only Maastricht University from the Netherlands had more than 200 publications, followed by KU Leuven (n = 125), the State University System of Florida (n = 102), Harvard University (n = 96), and the University of London (n = 90). In general, there is still room for exploration of kinesiophobia, and academic exchanges and collaboration among institutions should be strengthened.

### 3.3. Author co-occurrence

**Figure 4.**
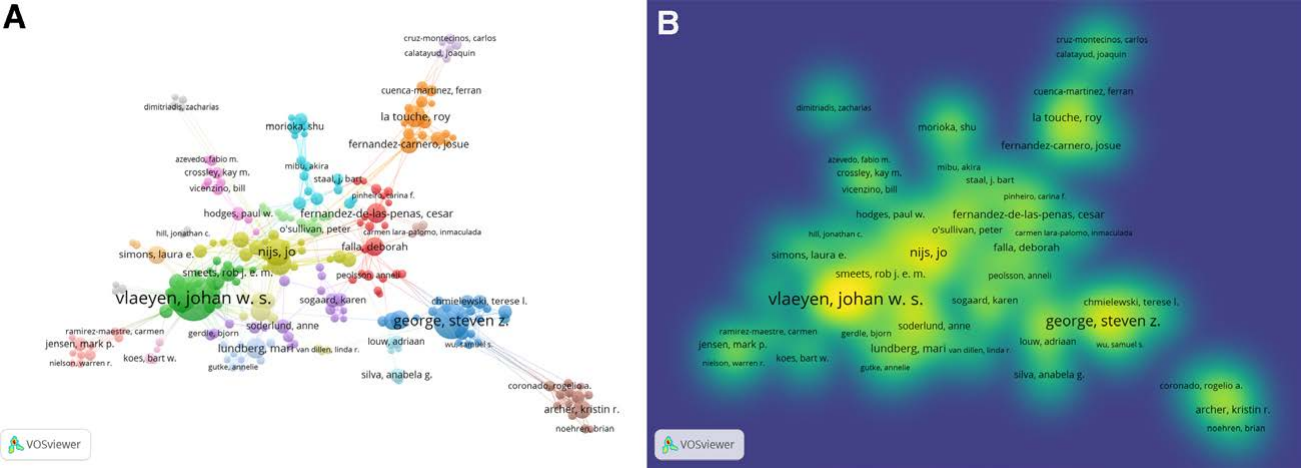
Author cooperation map. (A) Author co-occurrence network visualization. Nodes and their sizes represent authors and frequency of co-occurrence, and the lines between nodes represent the collaborative relationship between authors. Nodes and lines of the same color were treated as the same cluster. (B) Author co-occurrence density visualization. Each author label has a color indicating the density of that point, ranging from blue to green to yellow. The more co-occurrence frequency of an author, the closer the color around the author label is to yellow, and the higher the weight of its co-occurrence network relationship.

Price Law is an important reference standard used in bibliometrics to determine the core authors in a research field.^[20]^ Using this law, we defined the authors who published more than 39 articles in the field of kinesiophobia in the past 20 years as core authors. Vlaeyen JWS (102 documents, 7009 citations), George SZ (52 documents, 2605 citations), and Meulders A (41 documents, 935 citations) were the only core authors (within the last 20 years). These authors have published a considerable number of studies, which are widely cited and provide important reference and support for other scholars and future research. Table 1 lists the top 10 authors in terms of the number of publications in the research areas discussed in this article. The author co-occurrence map generated from VOSviewer (Fig. 4A) displays clusters of cooperative relationships based on author co-occurrence. This map clearly shows groups of authors with collaborations, such as the Vlaeyen JWS group, George SZ group, Sogaard K group, Nijs J group, and La Touche R group. The intersecting lines in the figure within and between clusters indicate that there is also some academic communication and collaboration among groups of authors, which is obviously beneficial for future research. The density visualization (Fig. 4B) clearly shows that the 3 teams led by Vlaeyen J.W.S., Nijs J., and George S.Z. had an important influence on the research and achievements in this field.

**Table 1:** Top 10 authors of articles related to kinesiophobia (2002–2022).

Rank	Author	Nationality	Document	Citation	H-index
1	Vlaeyen JWS	Belgium/Netherlands	102	7006	83
2	George SZ	United States	52	2605	58
3	Meulders A	Netherlands	41	935	25
4	Nijs J	Belgium	38	1036	62
5	Monticone M	Italy	30	788	28
6	Meeus M	Belgium	29	812	53
7	Sanford LD	United States	28	299	31
8	Rocca B	Italy	27	758	22
9	La Touche R	Spain	26	399	27
10	Sullivan MJL	Canada	25	1051	39

### 3.4. Journal and category distribution

**Figure 5.**
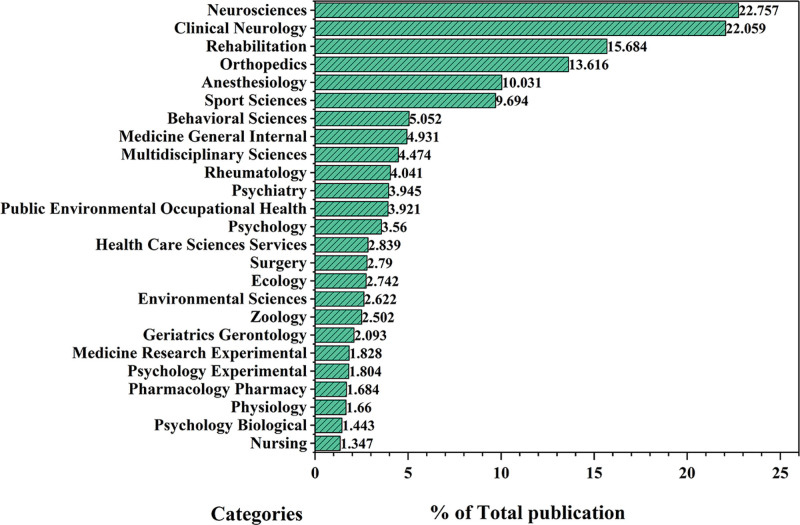
Category distributions.

Table 2 shows the top 15 journals in terms of publications in the field of kinesiophobia research in the last 20 years. *Pain* had the largest number of publications (n = 130, accounting for 3.127% of the total), followed by *European Journal of Pain* (n = 112, accounting for 2.694%) and *BMC Musculoskeletal Disorders* (n = 96, 2.309%). The journals with the top impact factors (IF) were *Pain* (IF = 7.4), *Scientific Reports* (IF = 4.6), and *Archives of Physical Medicine and Rehabilitation* (IF = 4.3). *PLoS One* (H-index = 404) had the highest H-index. This was followed by *Pain* (H-index = 282) and *Scientific Reports* (H-index = 282). Eleven of these journals were from the United States, and 6 were from the United Kingdom. According to the available information, the number of publications on kinesiophobia in journals is generally small, and many studies in this field were published in journals with low impact factors, which suggests that there is indeed room for future research on kinesiophobia. The distribution of the main categories of publications is shown in Figure 5. Research on kinesiophobia has been characterized by interdisciplinary and multidisciplinary integration, which is related to the complexity of the mechanism of kinesiophobia and its prevalence. The research fields mainly included neurosciences (22.757%), clinical neurology (22.059%), rehabilitation (15.684%), orthopedics (13.616%), clinical neurology (22.059%), anesthesiology (10.031%), and sport sciences (9.694%). Research on the occurrence, progression, and intervention of kinesiophobia is closely related to the physiological mechanisms (i.e., the field of neuroscience). Research in the field of neuroscience is the basis for understanding and dealing with kinesiophobia. Exercise is an effective means of rehabilitation after the acute stage of many diseases; thus, it is logical that rehabilitation researchers are interested in kinesiophobia.

**Table 2:** Top 15 journals with the most published articles on kinesiophobia.

Rank	Amount	% of total amount	Journal				
			Title	Country/region	Quartile (2022)	IF (2022)	H-index (2022)
1	130	3.127	*Pain*	USA	Q1	7.4	282
2	112	2.694	*European Journal of Pain*	England	Q2	3.6	121
3	96	2.309	*BMC Musculoskeletal Disorders*	England	Q2	2.3	112
4	94	2.261	*PLoS One*	USA	Q2	3.7	404
5	92	2.213	*Clinical Journal of Pain*	USA	Q2	2.9	138
6	86	2.069	*Journal of Pain*	USA	Q2	4.0	146
7	72	1.732	*Physical Therapy*	USA	Q1	3.2	169
8	71	1.708	*Spine*	USA	Q3	3.0	279
9	61	1.467	*International Journal of Environmental Research and Public Health*	Switzerland	/	/	/
10	59	1.419	*Disability and Rehabilitation*	England	Q2	2.2	124
11	51	1.227	*Archives of Physical Medicine and Rehabilitation*	USA	Q1	4.3	206
12	47	1.131	*Scientific Reports*	England	Q2	4.6	282
13	46	1.107	*European Spine Journal*	USA	Q3	2.8	155
14	46	1.107	*Physiotherapy Theory and Practice*	England	Q2	2.0	54
15	38	0.914	*BMJ Open*	England	Q2	2.9	139

/: The journal is not included in the latest SCI-E database.

H-index = high citation index, IF = impact factor.

### 3.5. Co-citation analysis

**Figure 6.**
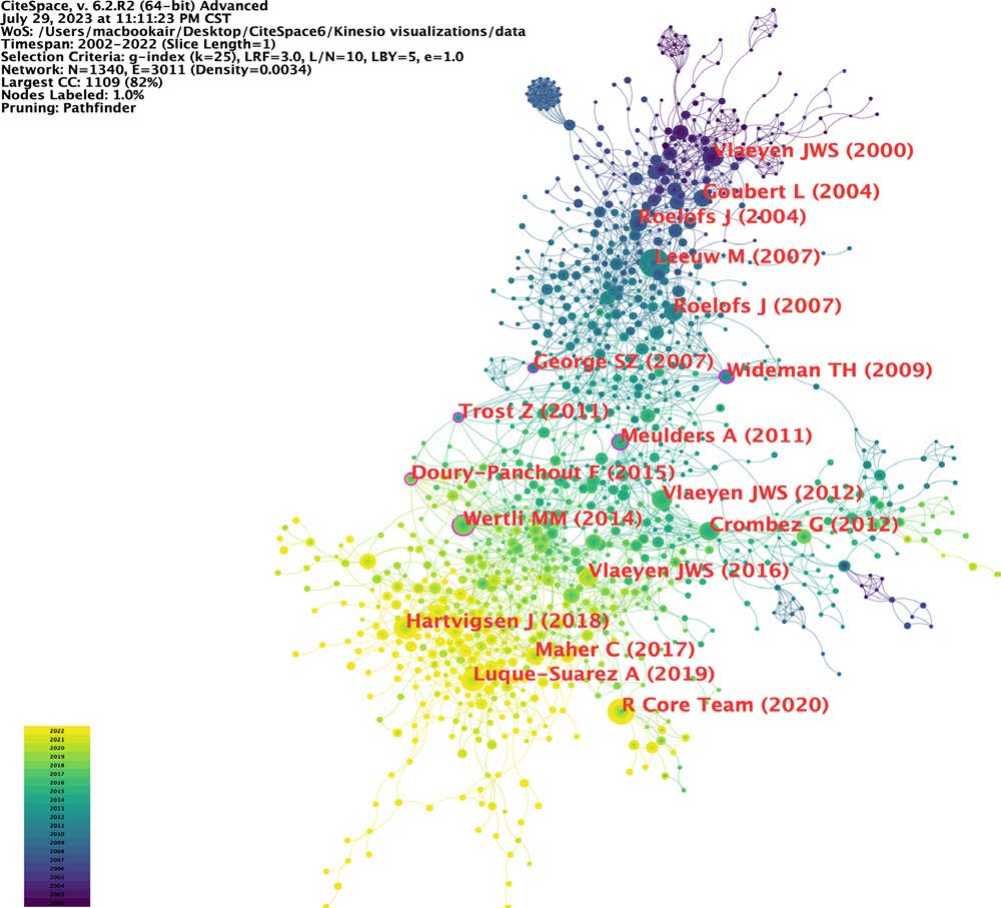
Knowledge map of co-citations. Articles are represented by nodes, and the line represents the co-citation relationship between the 2 articles. The darker the node, the older the article is. The nodes with the purple outer circle are strong centrality articles, which have important influence in their research field.

Articles with high co-citation frequency in the specified time period were considered to have an important impact on the research field under discussion in the time period of interest. These important articles, discovered through visualization, are useful for our following historical review. With the help of CiteSpace, we generated a co-citation map (Fig. 6) and extracted the top 15 articles and 6 articles with high centrality (Tables 3 and 4). The main topics of these articles were the fear-avoidance model or fear-avoidance belief,^[21–23]^ Tampa Scale for kinesiophobia,^[24,25]^ pain-related fear and disability,^[5,26]^ and intervention measures for kinesiophobia.^[27,28]^ Five articles were cited more than 50 times. The most frequently cited article was “The fear-avoidance model of musculoskeletal pain: Current state of scientific evidence.” This article^[21]^ reviewed the current status of scientific evidence for each component of the fear-avoidance model, emphasizing the role of pain-related fear in the initiation of low back pain, the development from acute to chronic low back pain, and the maintenance of persistent pain. Vlaeyen J.W.S., one of the core authors according to the previous analysis, had 4 papers on the list. The author review of the treatment of kinesiophobia^[28]^ discussed the difference between in vivo exposure and general graded activity, reporting a related trial. Finally, the authors observed improvements in pain-related fear and catastrophizing levels only during exposure treatment in patients.

**Table 3:** Top 15 most co-cited kinesiophobia related articles.

Rank	Co-cited number	The tittle of article	Yr	Author
1	105	The fear-avoidance model of musculoskeletal pain: current state of scientific evidence	2007	Leeuw M.
2	68	What low back pain is and why we need to pay attention	2018	Hartvigsen J.
3	67	Role of kinesiophobia on pain, disability and quality of life in people suffering from chronic musculoskeletal pain: a systematic review	2019	Luque-Suarez A.
4	53	Fear avoidance model of chronic musculoskeletal pain: 12 yr on	2012	Vlaeyen J.W.S.
5	52	Fear-avoidance and its consequences in chronic musculoskeletal pain: a state of the art	2000	Vlaeyen J.W.S.
6	48	The fear-avoidance model of pain	2016	Vlaeyen J.W.S.
7	48	Fear-avoidance model of chronic pain the next generation	2012	Crombez G.
8	47	The role of fear avoidance beliefs as a prognostic factor for outcome in patients with nonspecific low back pain: a systematic review	2014	Wertli M.M.
9	45	Fear of movement and (re)injury in chronic musculoskeletal pain: evidence for an invariant 2-factor model of the Tampa Scale for Kinesiophobia across pain diagnoses and Dutch, Swedish,and Canadian samples	2007	Roelofs J.
10	44	nonspecific low back pain	2017	Maher C.
11	43	Confirmatory factor analysis of the Tampa scale, for kinesiophobia-invariant 2-factor model across low back pain patients and fibromyalgia patients	2004	Goubert L.
12	42	The Tampa Scale for Kinesiophobia: further examination of psychometric properties in patients with chronic low back pain and fibromyalgia	2004	Roelofs J.
13	35	Exposure in vivo versus operant graded activity in chronic low back pain patients: Results of a randomized controlled trial	2008	Leeuw M
14	32	Fear-avoidance beliefs-a moderator of treatment efficacy in patients with low back pain: a systematic review	2014	Wertli M.M.
15	31	The treatment of fear of movement/(re)injury in chronic low back pain: Further evidence on the effectiveness of exposure in vivo	2002	Vlaeyen J.W.S.

**Table 4:** The 6 articles with the highest centrality.

Rank	Centrality	The title of article	Yr	Author
1	0.21	A prospective sequential analysis of the fear-avoidance model of pain	2009	Wideman T.H.
2	0.18	Pain-related fear and avoidance of physical exertion following delayed-onset muscle soreness	2011	Trost Z.
3	0.13	Fear of pain influences outcomes after exercise-induced delayed onset muscle soreness at the shoulder	2007	George S.Z.
4	0.12	The role of fear avoidance beliefs as a prognostic factor for outcome in patients with nonspecific low back pain: a systematic review	2014	Wertli M.M.
5	0.11	The acquisition of fear of movement-related pain and associative learning: a novel pain-relevant human fear conditioning paradigm	2011	Meulders A.
6	0.10	Kinesiophobia negatively influences recovery of joint function following total knee arthroplasty	2015	Doury-Panchout F.

### 3.6. Keywords co-occurrence and artificial clustering

**Figure 7.**
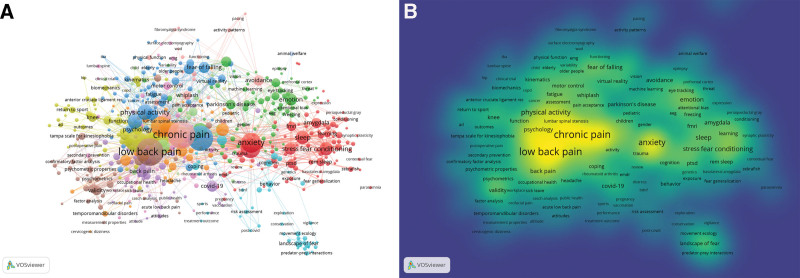
Mapping of keywords. (A) Keyword co-occurrence network visualization. The size and color of nodes represent the frequency and cluster of keywords, respectively. The line between nodes represents the co-occurrence relationship of 2 keywords. (B) Keyword co-occurrence density visualization. Each keyword label has a color representing the density of the point, ranging from blue to green to yellow. The more frequent the co-occurrence of a label, the closer the color around the label is to yellow, and the higher its weight.

**Figure 8.**
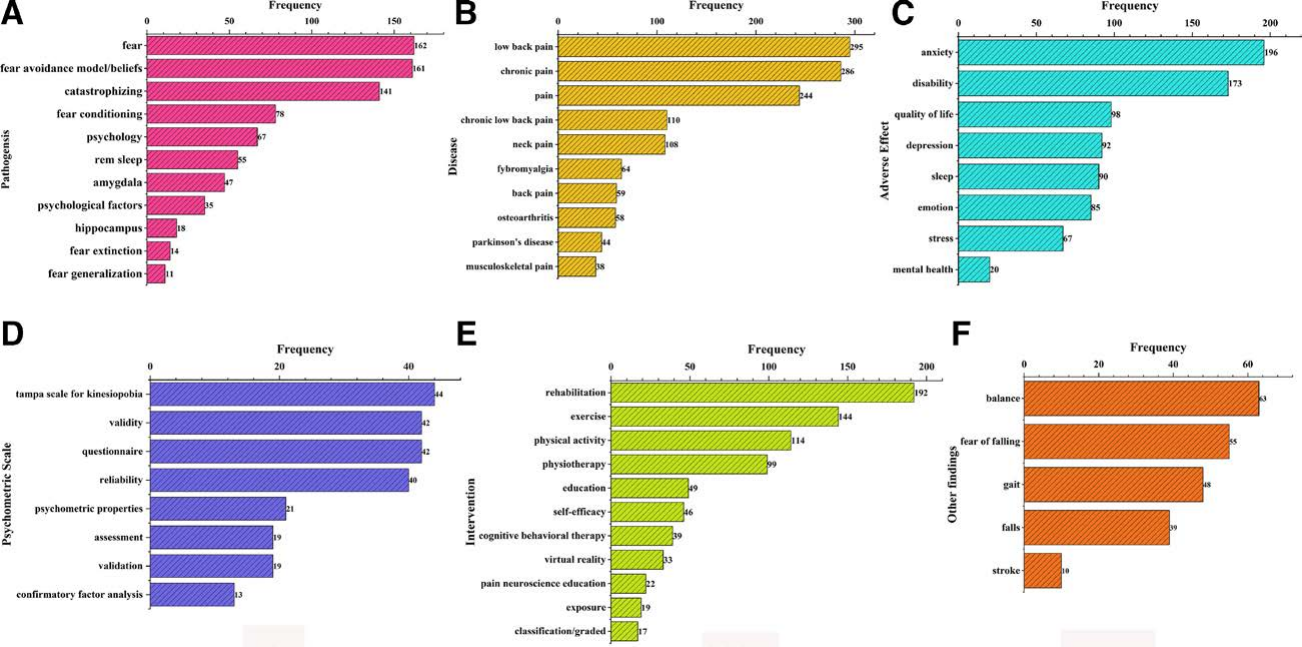
Artificial clustering results. (A) Pathogenesis. (B) Disease. (C) Adverse effect. (D) Psychometric scale. (E) Intervention. (F) Other findings.

Keywords are the distillation of the core content of an article, reflecting the focus of the article and the authors. Keyword cooccurrence analysis can indicate the hot spots and frontiers of a research field. VOSviewer was used to generate the keyword knowledge map (Fig. 7), and 534 keywords were included in the analysis. Kinesiophobia and fear of movement keywords repeated the search terms; these keywords were manually removed, and 532 keywords were finally analyzed. As shown in the figure, the node size reflects the frequency of the keyword, the lines represent the co-occurrence relationship, and the color indicates the keyword cluster according to the clustering algorithm. The most striking nodes in the figure (Fig. 7A) are low back pain, chronic pain, anxiety and physical activity, which indicates that these keywords have the highest co-occurrence and are the focus of this research topic. This may be related to the close relationship between fear-avoidance and fear of pain. The density visualization (Fig. 7B) also showed that the abovementioned 3 keywords had the highest density, suggesting that research related to pain and fear has been closely related for many years and accounts for a substantial proportion of research in this field. In the comprehensive analysis and discussion combined with a historical review, we manually clustered some high-frequency keywords and calculated associated statistics. The clustering results are shown in Figure 8. The 6 clusters were as follows: #1 pathogenesis, #2 associated diseases, #3 adverse effect, #4 psychometric scale, #5 intervention, and #6 other findings.

### 3.7. Keywords with citation bursts

**Figure 9.**
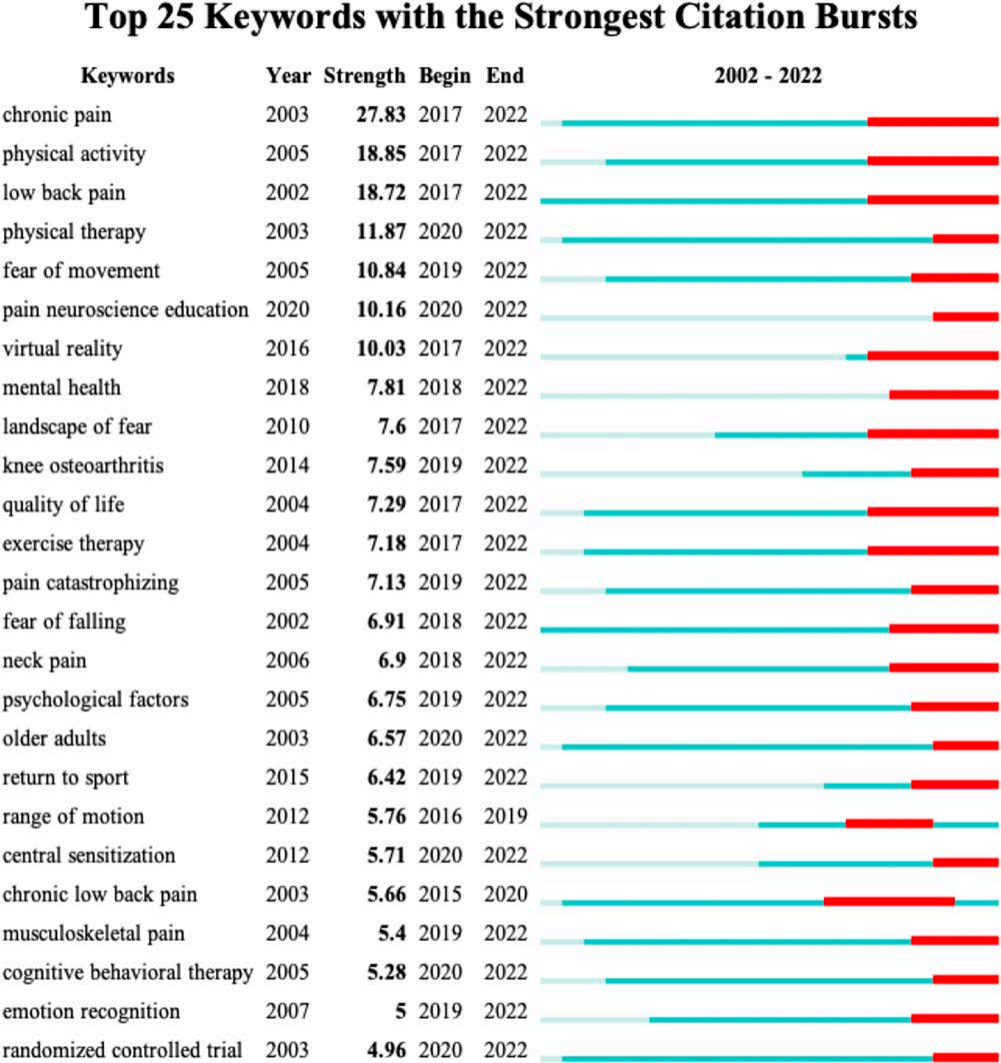
Keywords with the strongest citation bursts. Blue bars and red bars mean that some keywords are cited frequently in a certain period. Red bars mean the strongest citation burst, blue bars are weaker, and gray means the keyword is not cited or discussed.

CiteSpace can identify large changes in a certain variable over a specific time period through burst detection, and this fluctuation can be used to reflect deeper changes. By taking the “burstiness” keywords as the detection object, the research hotspots in a specific research field and the rise and fall of a specific research direction can be identified to a certain extent. Figure 9 shows the top 25 keywords with the most citations in the field of kinesiophobia research from 2002 to 2022. The red axis represents the period in which the bursts were strongest, and the gray axis represents the period in which the keywords were not detected. As seen in the figure, a large number of kinesiophobia-related topics have been discussed by researchers in recent years, kinesiophobia has begun to receive extensive attention, and more important and scientific conclusions are expected. In addition, chronic pain, physical activity, and low back pain were the top 3 keywords. It is worth noting that in recent years, several thematic directions with the strongest citation bursts immediately or soon after their emergence were pain neuroscience education, mental health, and virtual reality (VR).

## 4. Discussion

### 4.1. Basics

According to the bibliometrics results, research on kinesiophobia has steadily increased, with a large increase since 2020. Regarding the subject categories and publication types of kinesiophobia research, a wide number of subjects are related, which may be due to kinesiophobia complex pathogenesis, wide range of patients, profound impact on patients’ physical and psychological outcomes, and increasing attention to mental health. These ideas were verified by manual clustering of keywords. However, from the multidimensional analysis of the total number of publications; the distribution of countries, institutions, and journals; and the core authors in the past 20 years, the field of kinesiophobia still has space for exploration, and in-depth research is needed. The United States has conducted more research related to kinesiophobia than other countries, and Kori, who first proposed “kinesiophobia” is a neuroscientist from the United States. Well-known institutions such as Harvard University, the State University System of Florida, the University of California system and Harvard Medical School have been the main contributors to kinesiophobia research in the United States. In Europe, Masstricht University (Netherlands), KU Leuven (Belgium) and the University of London (England) are representative institutions. The University of Sydney and the University of Queensland in Australia are also representative institutions, while research in other regions may be lagging behind.

JWS Vlaeyen, PhD, full professor at KU Leuven and Maastricht University, is one of the scholars with important contributions to the study of kinesiophobia. His main research interests/expertise include the behavioral, cognitive and motivational mechanisms that transform common acute aversive feelings (pain) into chronic physical symptoms and disability.^[29,30]^ He and his team have examined the role of unpredictability in the generalization of somatic symptoms and disease behavior, with a particular focus on the competition between avoidance and reward-seeking tendencies.^[31]^ George, S.Z. from the United States also proposed hypotheses and carried out studies on the potential effects of fear avoidance or phobia, possible effective interventions, and measurement of psychological characteristics.^[32–34]^ However, one important question is why more studies on kinesiophobia have been published in journals with lower impact factors? There may still be a lot of work to do. As more researchers become interested in kinesiophobia, the situation is likely to be improved, and more findings will provide high-quality, convincing and scientific evidence for clinical practice. It is important not to ignore the academic exchanges and cooperation among countries/regions, institutions and scholars.

### 4.2. Artificial clustering results

#### 4.2.1. Pathogenesis and associated diseases.

To understand the mechanism of kinesiophobia, one must understand the fear avoidance model. In 1995, Vlaeyen et al^[3,35]^ established a cognitive-oriented pain-related fear model based on previous findings. The following discussion may help interpret this model. The negative evaluation of pain and its consequences is a precursor to pain-related fear. For example, pain catastrophizing is an exaggerated negative psychological response to harmful stimuli.^[36]^ People with high levels of negative emotions are more likely to be hyperalert to any threat and therefore more likely to develop fear. In addition, overestimation of pain may also make fear more likely. Fear of pain will affect physical performance, and avoidance behavior is one of the consequences, leading to reductions in patients’ daily activity. Long-term avoidance and inactivity will also affect the skeletal muscle and cardiovascular system, which may be result in symptoms of disuse, leading to worsening pain and disability. Another problem with avoidance is emotional disorders, such as depression and frustration, and negative emotions can indirectly affect avoidance by exacerbating the fear of pain. Patients are thus trapped in a vicious cycle of avoidance, disability, and increased pain.^[13]^

In addition, neurobiological studies of threat processing and fear memory have indicated new directions and are highly important for understanding the pathogenesis of fear and pain-related fear. Due to the plasticity and adaptability of the brain, previous traumatic or threatening experiences will make the brain respond faster to threats in the future.^[14]^ According to the cognitively oriented fear model mentioned above, judgments of potential danger may also come from false threat signals, and such responses may be maladaptive, such as avoidance behaviors or fear of activities. Threat signals are transmitted to the lateral amygdala after being perceived; subsequently, they are transmitted to the central nucleus and to the nucleus accumbens, after passing through the basal nucleus, thereby generating physiological, emotional, and behavioral responses related to fear. The posterior portion of the amygdala is involved in the descending pain regulatory system, which controls pain-related emotion, behavior, and motivation.^[37–39]^ Studies have found that areas such as the amygdala, insula, anterior cingulate cortex and hippocampus play an important role in fear acquisition, and the amygdala is also crucial for fear extinction and learning of safety.^[40,41]^ Meier et al^[42]^ found that phobic movement was positively correlated with the brain activity of fear-related brain areas such as the amygdala and insula. The differential effect of kinesiophobia in patients with chronic low back pain and pain-free subjects is reflected in the extended amygdala and its connection with the anterior insula. Genzel et al^[43]^ reviewed previous studies and found a large amount of evidence to support the effect of rapid eye movement sleep on amygdala-related memory processing; that is, the amygdala-hippocampal-medial prefrontal network involved in fear memory showed the strongest activity during rapid eye movement sleep. In general, the neurobiological mechanism of pain-related fear and kinesiophobia is extremely complex, and comprehensive research of the brain circuit and its synapses, neuronal interaction and physiological encoding of information will help us to develop new strategies for the prevention and treatment of kinesiophobia.^[14]^

#### 4.2.2. Adverse effects.

One of the main features of fear is the avoidance of perceived threats, leading to reductions in daily activities, poor performance, disability or muscle disuse. Numerous studies have confirmed that kinesiophobia is an important predictor of disability and psychological stress in patients with various types of pain that may impact their quality of life.^[5,6,35,44]^ Pells et al^[44]^ recruited 67 patients with sickle cell disease to evaluate and analyze kinesiophobia, pain, and psychological distress and found that patients with a higher severity of kinesiophobia had a higher degree of pain and psychopathology. This may be because the avoidance behavior and pain induced by kinesiophobia will reduce patients’ resources for coping with stress and thus lead to greater psychological distress. A study from Turkey examined the impact of kinesiophobia on lymphedema, upper limb function, depression/anxiety, and quality of life in breast cancer survivors.^[6]^ The study involved 81 breast cancer patients (age range, 44 to 70 years), all of whom had undergone cancer-related surgery within the previous 6 months. The results of the survey showed that 25 (30.8%) had kinesiophobia. In addition, kinesiophobia in breast cancer survivors increased the risk of depression/anxiety and decreased upper limb function. No correlation between kinesiophobia and quality of life was observed in this study, which may be due to the sample size and assessment tools. To explore the correlation of kinesiophobia with pain, disability and quality of life in patients with chronic musculoskeletal pain, Luque-Suarez et al^[5]^ conducted a systematic review of 63 studies with 10,726 participants and found that there was strong evidence of an association between the severity of kinesiophobia and pain intensity and disability as well as moderate-quality evidence that the severity of kinesiophobia is associated with lower quality of life and greater disability. Given that kinesiophobia in patients with various diagnoses of pain is more widely discussed, further scientific research on the adverse effects of kinesiophobia on the physical and psychological aspects of patients is needed.

#### 4.2.3. Psychometric scale.

The Tampa Scale for Kinesiophobia (TSK), developed in 1991, is a common measure of fear of movement/(re)injury.^[45]^ The original TSK consists of 17 items scored on a 4-point Likert scale ranging from 1 (strongly disagree) to 4 (strongly agree), with a total score ranging from 17 to 68. The higher the score is, the higher the patient fear of exercise. If the score exceeds 37, the patient is considered to have kinesiophobia. Later studies confirmed the validity and reliability of the TSK.^[46,47]^ Woby et al^[47]^ designed a short version of the TSK (the TSK-11) by removing 6 items with poor psychometric characteristics from the original version (4,8,9,12,14,16). The results in a sample of 149 patients with chronic low back pain showed that the TSK-11 had good internal consistency, responsiveness and construct validity equivalent to the TSK, while taking less time to complete. Subsequently, some scholars included data from 1109 patients with work-related upper limb diseases in confirmatory factor analysis, and the results showed that the 2-factor model of the TSK-11 consisting of somatic focus and activity avoidance had the best fit.^[24]^ This is consistent with the findings of Tkachuk and Harris.^[48]^ Therefore, the TSK-11 tends to be more recommended. Other measures of fear of movement/(re)injury include the Fear-Avoidance Beliefs Questionnaire,^[49]^ Kinesiophobia Causes Scale,^[4]^ and Fear-Avoidance Components Scale.^[50]^ Although the reliability and validity and cross-cultural adaptation of existing measurement tools had been extensively studied, most of these studies were based on low back pain, chronic skeletal muscle pain, or fibromyalgia.^[24,46,49,50]^ However, with the development of medicine, the study of kinesiophobia had long been associated with a variety of different types of pain-related diagnoses.^[8–12]^ We believe that special measurement tools for kinesiophobia for special diagnoses can be one of the directions of future research.

#### 4.2.4. Intervention.

Previous studies have found that pain education for patients with pain-related fear may promote behavior change, but actual experience is more persuasive than hearing this information only; patients with kinesiophobia may benefit from graded exposure to sports and activities they previously avoided.^[13]^ As kinesiophobia is the result of interactions among physiological, psychological and social factors, interventions are challenging as they need to adopt a multidisciplinary strategy addressing physiological and cognitive-behavioral aspects.^[51,52]^ Studies have proven that the multidisciplinary model, which combines psychological and cognitive aspects to help patients change their incorrect beliefs and perceptions and master effective health management methods, as well as a hierarchical activity plan and training courses led by therapists, can improve patients’ health outcomes by changing patients’ kinesiophobia beliefs, reducing pain catastrophizing and increasing pain self-efficacy.^[53,54]^ With the development of medical technology and interdisciplinary methods, more potentially effective methods have been discussed, such as exposure intervention based on behavioral observation,^[55]^ exposure intervention based on VR technology,^[56]^ eye movement desensitization and reprocessing therapy.^[57]^ On the other hand, more research and translation are needed.

#### 4.2.5. Other findings.

In the manual clustering of keywords, the appearance and combination of balance, falls, fear of falling and stroke attracted our attention. Subsequently, we reviewed much of the relevant literature and formulated a new perspective. Stroke is a disease that threatens human health, and more than 13.5 million people experience stroke every year. Most stroke patients have different degrees of dysfunction in language, movement and perception.^[58,59]^ Physical activity and rehabilitation exercise are effective ways for stroke patients to restore their physical function and improve their health outcomes.^[60]^ However, a review of the literature indicated that there are few studies on kinesiophobia in stroke survivors. Wasiuk-Zowada et al^[8]^ published a study on the occurrence of kinesiophobia in patients with neurological diseases in 2021; their study included 50 stroke patients, 81 multiple sclerosis patients and 61 Parkinson disease patients. Enrolled patients with stroke were generally in stable condition, were assessed at least 5 days after stroke onset, and had a modified Rankin Scale score of 2 or 3. The results showed that 128 people (66.67%) had severe kinesiophobia, and the incidence of kinesiophobia in stroke patients was close to 80%. In addition, Bąk et al^[61]^ conducted a cross-sectional study of 152 elderly patients with ischemic stroke in Poland, reporting that kinesiophobia was present in 78% of the patients. Sertel et al^[62]^ found that the balance score and fall efficacy score of stroke patients were negatively correlated with the severity of kinesiophobia. It is well known that fall and balance problems are the most common problems in stroke patients.^[63]^ Loss of balance in stroke patients weakens fall efficacy or the confidence that they will not fall. After developing a fear of falling, they will adopt escape strategies and reduce physical activity, resulting in a decline in physical function and balance confidence, which in turn will increase the risk of falling and the avoidance of exercise. Ultimately, it will hinder the functional recovery and the improvement in ability to independently perform daily activities of patients. Therefore, further research on kinesiophobia in stroke survivors is necessary and promising.

### 4.3. Hotspots and trends

According to the keyword co-occurrence and citation burst maps, chronic pain is the key research object and research hotspot of kinesiophobia, especially low back pain. This suggests that it is important to investigate and synthesize relevant evidence. The topic of chronic pain and kinesiophobia has been studied for a long time. Initially, the “fear-avoidance” model was proposed to explain the cause and process by which musculoskeletal pain developed into chronic pain syndrome in patients.^[1]^ The maintenance and exacerbation of the fear state is associated with an exaggerated perception of pain. Pain-related fear can lead to negative coping or avoidance behaviors,^[64,65]^ which may eventually cause related functional disuse or disability.^[30,66]^ Studies have found that 56%–72% of chronic pain patients are affected by kinesiophobia,^[67,68]^ and most of the scientific work on the treatment of kinesiophobia has focused on chronic pain disorders, especially low back pain and neck pain.^[51]^ This is consistent with the results of our bibliometric analysis. At present, research on kinesiophobia has extended beyond low back pain to other diseases associated with chronic pain or the development of chronic pain due to kinesiophobia, such as fibromyalgia, osteoarthritis, Parkinson disease, and even stroke and postoperative recovery.^[8,69,70]^

Further exploration of keywords with citations can identify possible future research trends. Pain neuroscience education, mental health and VR research were late to emerge but quickly resulted in a super-strong citation burst. This suggests that these 3 topics may be the research frontier in this field in the next few years. First, with the development of society, people have higher requirements for health care. Health involves not only good physical condition but also good mental health and quality of life. The “bio-psycho-social” medical model proposes that diseases should be understood from the biological level to the psychological and social levels to identify the determinants of diseases, provide a reasonable treatment, and develop a health care model.^[71]^ Kinesiophobia involves not only a simple fear of exercise but also a complex and multifactorial mindset derived from the belief of vulnerability.^[2]^ It is one of the potential psychological factors leading to the deterioration of patients’ physical function and is also closely related to mental health disorders, which seriously affect the rehabilitation outcomes and quality of life of patients. Therefore, the identification and intervention of kinesiophobia is an important project and challenge in the field of mental health. Since avoidance behaviors caused by fear occur in anticipation of pain, excessive anticipation of pain, catastrophizing and related negative beliefs need to be transformed.^[13]^ Pain neuroscience education (PNE) is an effective way to alter patients’ pain beliefs and deepen their understanding of pain by educating them about neurobiology, neurophysiology, pain processes and performance. Finally, this method ensures that the patient understands that the movement or exercise that causes pain is safe and that the patient has the confidence to perform the activity.^[72,73]^ A meta-analysis showed that the combination of PNE and exercise for chronic musculoskeletal pain resulted in greater short-term improvements in pain, disability, kinesiophobia, and pain catastrophizing compared with exercise alone.^[74]^ From the psychological and physiological aspects, PNE is an effective psychotherapy, and the combination of PNE and various forms of physical therapy as a treatment for kinesiophobia is worth exploring.^[51]^ Similarly, sex, age, and education level may be associated with kinesiophobia severity; thus, the diverse and personalized PNE method may become an option with future research.^[8,75]^ Another promising approach is VR. In 2017, Yelvar published a study on the short-term efficacy of VR combined with physical therapy on pain, function and kinesiophobia in patients with subacute and chronic nonspecific low back pain and reported positive experimental results.^[76]^ Gulsen et al^[56]^ also showed that compared with simple exercise therapy, exercise training combined with VR led to significant improvements in patients’ pain, kinesiophobia and mental quality of life. Previous studies have reached similar conclusions. A 2013 meta-analysis of 11 randomized controlled trials involving 488 subjects showed that the use of VR technology can reduce the severity of kinesiophobia in patients with chronic low back pain.^[77]^ The study also found that nonimmersive VR was more effective than fully immersive VR. VR combined with exercise was more effective than VR alone.^[77]^ VR technology has potential therapeutic applications for kinesiophobia, but future studies containing more details, such as specific scenario designs for different diagnoses, details of treatment, safety, and supervision, are needed.

## 5. Conclusions

In this study, the bibliometric tools CiteSpace and VOSviewer were used to analyze studies on kinesiophobia included in the WOSCC and published in the past 20 years. Cooccurrence analysis was conducted on the countries/regions, institutions, authors and keywords in this field to understand the basic knowledge structure of research in this field. Second, artificial clustering based on keyword cooccurrence was conducted; the literature was reviewed to summarize the pathogenesis, adverse effects, measurement tools and intervention strategies of kinesiophobia; and potential research directions were proposed. Finally, combined with previous work, co-citations and keyword bursts, we objectively and comprehensively summarized research hotspots and trends. We believe that this study can provide an important reference for future scientific work on kinesiophobia.

### 5.1. Limitations

It is clearly a limitation that the data in this study were derived only from the SCI-E database of the WOSCC, which prevented us from considering the full context of this research field. However, the SCI-E contains a large amount of kinesiophobia-related research data, ensuring the quality of the literature, and our results are still scientifically reliable. Next, we will learn to retrieve and export data from more databases, to improve this shortcoming and to compare analytic results.

## Acknowledgments

We would like to thank the editor and anonymous reviewers for their helpful comments on early versions of this manuscript.

## Author contributions

**Conceptualization:** Linzhang Li, Yan Sun.

**Data curation:** Hua Qin.

**Formal analysis:** Linzhang Li, Jun Zhou, Xiaojuan Yang.

**Funding acquisition:** Yan Sun.

**Methodology:** Linzhang Li, Aiying Li, Jin Zhang.

**Supervision:** Yan Sun.

**Validation:** Linzhang Li.

**Visualization:** Linzhang Li, Yan Zhang.

**Writing – original draft:** Linzhang Li, Hua Qin.

**Writing – review & editing:** Aiying Li, Jin Zhang.
